# Mechanisms in biomedical ontology

**DOI:** 10.1186/2041-1480-3-S2-S9

**Published:** 2012-09-21

**Authors:** Johannes Röhl

**Affiliations:** 1 Department of Philosophy, University of Rostock, August-Bebel-Strasse 28, 18051 Rostock, Germany

## Abstract

The concept of a mechanism has become a standard proposal for explanations in biology. It has been claimed that mechanistic explanations are appropriate for systems biology, because they occupy a middle ground between strict reductionism and holism. Because of their importance in the field a formal ontological description of mechanisms is desirable. The standard philosophical accounts of mechanisms are often ambiguous and lack the clarity that can be provided by a formal-ontological framework. The goal of this paper is to clarify some of these ambiguities and suggest such a framework for mechanisms. Taking some hints from an "ontology of devices" I suggest as a general approach for this task the introduction of functional kinds and functional parts by which the particular relations between a mechanism and its components can be captured.

## Background

The concept of a mechanism has in recent years become a standard philosophical proposal for explanations in biology and other sciences of complex systems where the traditional approach, subsumption under universal laws, has not been fruitful. This is in agreement with the practice of these sciences where the postulation of mechanisms on several levels (organismic, cellular, molecular, biochemical) is a common research practice: A stable behaviour of some biological system is explained by the description of a (postulated) mechanism that is causally responsible for this behaviour [1-5]. Familiar examples include photosynthesis or protein biosynthesis; diseases are associated with mechanisms as well as the action of drugs [6]. The actual discovery of such a postulated mechanism is of course non-trivial and often a seminal scientific achievement. Whereas mechanistic explanations in biology have usually been mostly qualitative, Systems Biology is working with powerful mathematical tools and striving for quantitative results. Scientists working in the field have expressed the hope to get a better picture of biological reality if the computational approach could be aligned with mechanistic approaches [7]. The philosophical discussions of mechanisms often focus on explanation, not ontology, and suffer from ambiguities and lack of clarity that could be amended by a more formal approach. The following considerations hope to give some first steps in this direction by an ontological analysis of mechanisms. Though the work presented here is conceptual groundwork, there is a rich field of possible applications in biomedical knowledge representation and knowledge eliciting.

Ontology should answer the question into which fundamental category something falls. To find out what a mechanism is, I will start with some definitions from the literature, discuss options for categories for the components of mechanisms and the mechanism as a whole, and suggest with which relations they could be tied together to constitute a mechanism. It should be noted beforehand, however, that the suggestions in this paper are not meant to necessarily imply a universal mechanistic and reductionist world view. Different authors use somewhat different notions of "mechanism" or "mechanistic" depending on their respective background in biology, philosophy, physics or information science. It will be clarified in the following that mechanism in the sense used here is dependent on functional concepts and thus not reductionist in the sense criticized by Robert Rosen [8,9]. Rosen contrasts mechanistic causation with biological final causation. In his view a mechanistic system is characterized by deterministic and computable behavior, that is any full description of a state of the system together with the mathematical operator for time evolution or the system's equations of motion determines the system states at later times. These systems can also be captured completely by a reductionist analysis of their components, i.e. the behaviour of the whole is completely determined by the behaviour of its parts ("bottom up" causation). On the contrary, certain types of systems such as biological ones are, according to Rosen, distinguished by the fact that they cannot be described adequately in "purely mechanistic" terms, because they exhibit features like the whole determining the behaviour of its parts ("top down causation"). An appraisal of Rosen's criticism and his very general formal distinctions between mechanistic (in the narrow sense used by him) and biological systems and their modes of causation has to be left for another occasion as the very possibility of top-down causation is a hotly contested topic in the philosophy of science. In any case, many proponents of mechanisms in the philosophy of biology seem to take their position as intermediary between "hard reductionism" (like the one criticized by Rosen as "mechanistic") and holism [10]. Furthermore, the claim that organisms contain (many) mechanisms is not meant to imply that all organisms are to be identified with mechanisms. An ontology that recognizes some important biological entities as mechanisms does not imply a universal mechanistic ontology of biology (or the whole world).

### What is a mechanism? - some definitions

Here are three different definitions for mechanisms from the literature on philosophy of biology:

"A mechanism for a behavior is a complex system that produces that behavior by the interaction of a number of parts, where the interactions between parts can be characterized by direct, change-relating generalizations." [2]

"Mechanisms are entities and activities organized such that they are productive of regular changes from start or set-up to finish or terminating conditions." [1]

"A mechanism is a structure performing a function in virtue of its component parts, component operations, and their organization. The orchestrated functioning of the mechanism is responsible for one or more phenomena." [3]

All three conceptions identify several features which are important for the characterization of mechanisms: Mechanisms are (1) for a specific behaviour, which can be characterized *functionally *by a specific input and output; they are (2) not merely stating such input-output-correlations, but show the "inner workings" producing the output; they exhibit (3) a kind of continuity and lead without gaps from initial conditions to final states, and they are (4) complex structures and may, in general, have submechanisms at a lower level of granularity.

The first two definitions have difficulties dealing with cyclical mechanisms that cannot be simply characterized by initial or final states [4]. This can be taken care of, if one thinks of the function performed by a mechanism in a more general way: A cyclical mechanism for maintaining some balance of concentrations, for example, will take as input any state of the system to be controlled, and if the value of the concentration ratio of this system is not within some tolerance interval, the mechanism will produce as output a state with a value within the interval. So the input/output states do not have to be thought of as temporally strictly ordered initial and final states. The key features seem to be that mechanisms are on the one hand functionally characterized, and on the other hand they are complex systems and the consecutive actions of their components together realize the function of the whole.

Furthermore, it could be asked, whether and on which grounds there is a distinction between mechanisms and biochemical "pathways" as the usage of these terms in the scientific community seems almost interchangeable. One difference seems to be that pathways are mostly chains of chemical reactions and transport phenomena involving freely floating biomolecules, whereas mechanisms are more clearly localized and involve stable material structures. For now I will assume that the mechanisms considered here deal with stable and localized structures. An expansion to systems that are not characterised by stable individual continuants, but by concentrations of "stuffs" that vary in time has to be left for another occasion. But I assume that the approach sketched below is sufficiently general to be extendable to pathways in the latter sense.

## Methods

I will first propose an ontological analysis of mechanisms and appropriate categories for their components, then look into the relationships and connections between them, and finally discuss the possible category choices for the mechanism as a whole. For that purpose something will have to be said about functions and functional parts.

### Components of a mechanism

According to the definitions above mechanisms have two types of components or parts: "entities" and "interactions" or "activities". An expression like "entity" is too general to be taken seriously as a suggestion for an ontological category (because usually everything that exists is taken to be an entity of some sorts). What these authors apparently mean with "entities" in the definitions above are stable material parts of the mechanism. According to the top level ontology BFO [11] which we will take as our ontological framework, these can be classified as independent continuants. (In the DOLCE ontology they would be called "physical endurants" [12].) In BFO independent continuant entities are what we would think of as "things", i.e., entities that can change in time, that is, they can exhibit different properties or states at different points in time, while retaining their identity, but do not have temporal parts. In contrast, occurrents (DOLCE: "perdurants") are entities like events or processes that take place in time and have temporal parts (phases or stages). Occurrents are dependent entities; they always need at least one independent continuant to participate in them. (A running process is for its existence dependent on least one runner enacting this running process.)

The material components of a mechanism are identified functionally according to their contribution for the behaviour characteristic of that mechanism as a whole. (More about functional contributions will be said below.) They are parts of the mechanism, but this is not sufficient, as not all parts of a mechanism are recognized as salient components. This is dependent on context, usually the context of the very mechanism in question. It is not just a question of parthood and granularity. E.g., the „pumped" H+-ions (protons) or the electrons transported by the electron transport chain in photosynthesis are components of the respective mechanisms (submechanisms of photosynthesis), because they contribute to the action of these mechanisms. But the protons and electrons of the atoms of the molecules that are not involved and do not contribute to the action of the mechanism are not components, although of course material parts (by transitivity of the parthood relation) of the whole material conglomerate. What about the other type of components and their relationship to the material components?

### Activities and interactions - the dynamics of a mechanism

Obviously the material components of a mechanism must be dynamically connected and integrated into the whole that is to be captured under the description of a mechanism. There is a dispute how this should be reflected by the ontological analysis. One option is to understand the interactions as relations between the continuants as proposed by Stuart Glennan: An "interaction" is taken to be a "correlative property change", "an occasion on which a change in a property of one part brings about a change in a property of another part" [2]. These correlations would be mere relations between the material components and their properties, and "interactions" would therefore not be entities in the narrower sense of a fundamental ontological category. To avoid regress the "interaction relation" between the components has to be immediate, i.e. the interactions must not be mediated by further intermediary components. On the other hand one can argue as Darden, Machamer and Craver do that to represent the dynamics of a mechanism it is not enough to use only the material components and their relations, but opt for the acceptance of "activities" as a separate class of entities in their own right: "Activities are the producers of change. Entities are the things that engage in activities. [...] An activity is usually designated by a verb or verb form [...]. They are constitutive of the transformations that yield new states of affairs or new products." [1]

According to this position, interactions would merely record the result of transitions between different states of the material components whereas activities would give a better, more complete description. Causal efficacy could only be ascribed to the material components if their specific activities were taken into account. Furthermore, the continuity and regularity of the temporal phases of an active mechanism could only be guaranteed by the acceptance of activities because they connect the states of the material components and thus assure a "productive continuity" between these phases. Machamer et al. claim that the dynamic behaviour of mechanisms would be captured better with activities as either themselves changes or producers of change than with only the results of changes (Glennans "interactions"). So according to Glennan interactions are mere relations dependent on their relata, they are not to be "reified" and do not form an additional ontological category. According to Machamer et al. activities are some kind of processes in which material components take part. If we accept those additional elements in our ontology we can classify "activities" as processual entities with temporal parts or phases, that is as occurents in the terminology above (DOLCE: perdurants).

James Tabery has suggested an intermediate position [13], accepting both interactions and activities as complementary to each other. Tabery starts with Glennan's conception of interactions as "occasion[s] on which a change in a property of one part brings about a change in a property of another part" [13]. This "bringing about" could be analysed further in terms of activities. An activity determines more precisely how such a correlative change is achieved: "*For the dualists, the activity is the dynamic process of bringing about*" [13]. But interactions are necessary, too, because there are productive as well as non-productive activities. The latter do not involve another interacting entity, e.g. changes of the geometrical conformations that only concern one molecule. Therefore, so Tabery, interactions are needed to distinguish productive from non-productive activities. To integrate activities into Glennan's account Tabery simply replaces in the definition of an interaction "brings about" by "dynamically produces": „an occasion on which a change in a property of one part *dynamically produces *a change in a property of another part" [13] (italics added). And activities are responsible for the dynamical production. The ontological implications of Tabery's suggestion are not completely clear as the relation between activities and interactions is not explained. One could understand activities as "realisers" of interactions: interactions would just record the mere correlations of property instances of two or more particulars and activities would contribute additional information about the course of the change. With a different reading the class of interactions could be taken to have two subclasses, "nonproductive" and "productive" interactions, and activities would then be the productive subtype of interactions. In any case, if Tabery's proposal is taken ontologically serious, it speaks against taking interactions as mere relations and in favour of accepting an additional ontological category of dynamic, occurent entities like the activities mentioned above.

Besides capturing the notion of dynamicity there are further advantages to an approach that accepts not only continuants and their relations, but also processes or occurrents. Current top level ontologies like BFO and DOLCE all recognize this need for occurrents. The continuants involved in the interaction are connected to occurrents by a relation like "participates_in" that expresses the involvement of one or more continuants in a process. By this "reification" of interaction processes one avoids having to deal with relations with different arities, if an interaction type can have tokens with different numbers of participants. (It should also be noted that in description logics like OWL DL relations with more than two relata are difficult to model. With a top level ontology that includes processes this restriction can often be worked around in a intuitively appealing way.) And with occurrents one can model the location, frequency and other interesting features of these interaction processes or facts about their preceding or following each other and define corresponding relations.(Cf. [14] for detailed arguments for the usefulness of admitting occurrents as a distinct category for representing molecular interactions.)

A note on the sometimes confusing terminology: In the Gene Ontology [15] the term "activity" is used to describe molecular *functions*, like "catalytic activity". In the GO this means that the specific component has the function (or disposition) to initiate or take part in this activity, not that activity, that is, the actual contribution to the process of a chemical reaction, itself. Whereas in the discussion summarized above and throughout this paper, "activities" are used as the processes within the structure of a mechanism. I will usually call interactions "processes" and ignore the difference to processes that involve only one participant and are therefore not interactions in the strict sense.

So as a first result we can conclude that the material components of mechanisms (like ions, cell membranes etc.) can be classified as continuants. Furthermore there are "processual components", that is the actual interaction processes going on in a mechanism (transporting of ions, energetic exciting of electrons etc.). These basic types of components are connected by relations like participation. Before discussing these relations between the material components and processes of a mechanism in more detail, I will turn to the question how the mechanism as a whole should be ontologically classified. For this purpose I will first describe an approach that suggests a very strong connection between a material structure and specific processes it can be involved in.

### The device ontology

A very general framework to analyze the mutual dependence of continuants, functions and processes has been suggested by Antony Galton and Riichiro Mizoguchi [16]. This has been developed for applications in the ontology of engineering, but is sufficiently general for our purposes. Starting from the analysis of engineering devices in their "ontology of devices" not only artefacts, but almost any kind of material entity can be considered as a "device" in the following general sense: A device is an object that is essentially characterized (functionally) by the input/output it can produce (apart from that it is a "black box"). But to generate that output the object has to undergo or "enact" a process: "an object [...] is characterized in terms of the processes it enacts. These are what we call the external processes or behaviour of the object" [16]. We thus have a tightly interrelated triple of <object, function, process>: The object "enacts" its "external processes", which produce "the generation of output from input" [16]. E.g., in photosynthesis, the object "photosystem I" has the function to transform the input light energy into the output chemical energy. If we look inside the "black box" we find "subdevices" with specific processes that are internal to the higher-level device and causally responsible for its external processes. And on any level the identity of an object depends on its function or disposition for external processes: "an object is a unity which is what enacts its external processes" [16]. Such a nested structure of objects essentially characterized by their functions or their ability to enact specific processes corresponds very well to the mechanistic approach in biology as described so far. The regress of structures (devices within devices) has to be stopped at some basic physical level, but this is not important for the purpose here. For biological mechanisms there will always be some ground level of chemical or physical entities and their interactions.

### The mechanism as a whole

There are conflicting intuitions into which category a mechanism as a whole should belong. Glennan thinks that it should be thought of as a continuant; a mechanism is a "thing" like a clock or a cell, because of its endurance and stability compared to a process or chain of events [2]. Similarly, Bechtel: "it consists of an arrangement of parts and has at least some enduring identity" [5]. On the other hand the mechanism could be thought of as a process. It should be noted that from the fact that a mechanism has processes as "components" it does not follow immediately that the mechanism has to be a process itself, because not only a process can contain processes (cf. the "device ontology"). The processes do not have to be considered parts in the usual sense, but containment can mean that process tokens have locations within the boundaries of the continuant. But it seems clear that the unfolding activity of a mechanism has temporal stages characterized by the sequence of processes its components enact. (In fact, this was used above as a motivation for the acceptance of internal processes instead of mere correlations.) And in our top level ontology BFO the only entities with such an explicit temporal structure are occurrents. So it might seem that a mechanism as a whole has to be a process. But what if a mechanism is not active? It seems that the mechanism is still in existence as a mechanism without any (external) process going on (like the case of a stopped clock that needs to be wound up). So if we want to recognize mechanisms as entities that persist as mechanisms even while they are not active, the mechanism cannot be categorized as a process.

If we take the mechanism as a generalized "device" in the sense of Galton and Mizoguchi, we might be able to do justice to both intuitions. We take the mechanism to be an independent continuant. It has a structure and parts, but it is also sufficiently unified and delimited that, as a whole, it can be considered as a kind of continuant. But it is functionally characterized, that means its very identity as a mechanism depends on its function for its specific process. In photosynthesis, the process of the conversion of light energy to chemical energy sets all the structures and material parts apart that contribute to this function and unifies them into the "thing" biologists call the photosynthesis mechanism. This close connection and mutual dependency of continuant, specific function and process also explains the initial uncertainty and conflicting intuitions about the appropriate category for a mechanism. Following the idea of the ontology of devices this can be expressed by the ascription of a specific function as essential for the mechanism. Mechanisms are then continuants and classified as functional kinds. If the function is an essential feature of a mechanism this would imply that the latter would cease to exist as such, should it lose the ability to perform its function. This seems rather strong for some cases, because we would usually classify a broken device or mechanism that has for a while lost its essential function still as an instance of the respective functional kind, e.g. a car that has to be repaired to be able to perform its usual function again, still is car. We would not say that the broken down car ceases to exist as a car and later comes into being again after having been repaired. There is some flexibility, probably domain dependent, how far beyond repair or recovery with respect to the exercise of its function something would have to be, to not longer be classified as a specific mechanism for this function.

### Functions and dispositions

So it turns out that a further element is necessary for the ontological connection of continuants with processes and has to be elucidated: functions and dispositions. Authors in the debate acknowledge that the characteristic processes of mechanisms and their components should be based on specific properties of the continuant components of the mechanism [1] and admit explicitly or implicitly the necessity of dispositions, so Glennan: "[T]he systems as a whole have stable dispositions - the behavior of these mechanisms" [2] and Tabery: "The interaction, then, is the event whereby one part induces a property change on another part by virtue of its own change-relating capability" [13]. The latter is just an awkward description of the connection between dispositional properties of the components and their interaction in virtue of theses dispositions. Care should be taken not to confuse the disposition and the process it is a disposition for (as seems to be the case in the quotation from Glennan). What kinds of entities are we dealing with? A common philosophical position takes dispositions as a type of properties [17]. The central point is that dispositions are essentially related to a process of realization. Something is water-soluble if it dissolves when put in water. In this fashion dispositions establish a link between continuants and processes and the fundamental connection is the following: *Continuant S has disposition D for realization P and S, the bearer of the disposition, is a participant in this process P*. In formal ontology, a suggestion is to treat dispositions as a kind of dependent continuants (in this respect analogous to qualities) and express the link to a process of realization by calling them "realizables" [18], see also [19] and [20] for more on this topic.

The concept of a function has been even more widely and controversially discussed in the philosophy of biology and elsewhere (cf. [21] and [22] for recent contributions to this debate, see also [23], chapters 5 and 14). An elaborate formal ontology of functions has been proposed in [24]. Both for lack of space and because I do not want to presuppose particular accounts of functions and functional roles, I will rely only on very general features of the concept of a function (apparently shared by Burek et al. [24], although their account contains more detail). Among the different approaches to functions in the philosophical debate two are of particular interest here: The "causal role analysis" and the "goal contribution analysis" (summarized by its main proponent Christopher Boorse in [25] which also contains further references). The former account is very straightforward and close to the way I have used "functional" so far in this paper: That × has function f simply means that × has the disposition to causally contribute to some output o of a complex system s. A well-known problem is that this account is extremely broad, actually too broad and admits many unintuitive "functions", e.g. that clouds should be ascribed the "function" to produce rain, because they undoubtedly have a central causal role in the production of rain (this and more examples and further criticism in [25]), whereas the usual sense of functions is connected with some intention as in artefactual functions or a (not necessarily intended or conscious) goal in biological functions. Boorse's goal contribution approach takes this feature into account: "X performs function Z in the G-ing of S at t if and only if at t, the Z-ing of × is a causal contribution to G" [25]. This is still a rather weak definition, because some functions could be performed only in one single case and fulfil it accidentally [25]. A stronger account can capture what is usually meant, if we distinguish between proper functions and accidental functions, which will be elucidated below.

In the top level ontology BFO functions, like dispositions (and roles) are "realizable dependent continuants" [18]. That means a function is dependent (like a property) on the independent continuant that is the bearer of the function. "Realizable" means as with dispositions that the instances of a function type are connected to processes, their realizations. These are processes with the bearer of the function as a participant. So we can translate Boorse's definition above into our terminology: Take × to belong to the class of continuants and be a part of a mechanism, then (the system) S is a mechanism and Z is a function. G would be the goal, that is a final state of the complete mechanism, "G-ing" is the process the mechanism enacts and "Z-ing" is the realization process of the function Z. (Below I will not distinguish further between "G" as a goal state and "G-ing" as process, but speak only of the realization process.)

A few remarks are in order here: Not all processes involving the bearer of the function are realizations of (one of) its functions, but every realization of the function involves its bearer as participant. If I drop a watch and it breaks, this breaking is a process involving the watch, but obviously not a process that counts as a realization of the function of the watch. A standard example is the concomitant production of noise made by the heart when it realizes its proper function to pump blood. It is a process caused by the action of the heart, but it is not the proper function of the heart to make that noise. And functions do not have to be (always or ever) realized, as e.g. in the case of a safety mechanism the function of which will only be realized if certain conditions obtain (and they may never obtain). I will ignore the complications of this modal feature for now. Dispositions are very similar in this last respect and are discussed at some length in [20].

About the relation of functions and dispositions the following can be said: both share the feature that they are "realizables" [18,19], that is they are essentially connected to a realization process, but that process will only take place if certain conditions are met. A difference between functions and dispositions lies in their context-dependence. Dispositions are inhering properties that do not depend on context. Continuants may lose or acquire dispositions, but many of them are not easily changed without fundamental changes to the bearer. In contrast, most functions can be performed by different types of bearers and an object may have different functions in different contexts without any change in itself. In my view, functions are based on dispositions, because the dispositions of a thing determine whether it can fulfil the respective function in a given context at all. (Electrons can only have the function to build a gradient in the electron transport chain because of their disposition charge.) This context-dependence is also connected with the problem of the distinction between proper and accidental function. To illustrate this with an example from the domain of artefacts: One function of a hammer is to drive in nails, a process token of hammering in one particular nail is a realization token of this function of this hammer, and the hammer is of course a participant in this process. Therefore a necessary condition for being a hammer is that it can participate in nailing processes which are realizations of its function. For a full definition one could introduce the intention that the hammer has been designed for this particular proper function. But a nail may also be driven in with help of a stone, so the stone could also perform the "hammering function" to some extent. According to the causal role and the goal contribution accounts in such a case this stone then has the function to drive in a nail. But hammering is not an essential function of the stone, rather an accidental one. This is different with many biological functions as these are not designed by intentions, but have evolved naturally. Still, we may draw the distinction between proper function and accidental function. We can ameliorate the problem of context-dependence and proper vs. accidental function in taking the mechanism in question as the relevant context that delimits the proper functions of its parts. We are not interested in any possible accidental function or in any possible context, but only in specific functions of parts within a specific mechanism. With this account of functions we can now define the class of functional kinds.

## Results and discussion

### Formal relations for the representation of mechanisms

I follow the suggestions of Smith et al. [26] and regard as primitive the particular level relations **instance_of **(which holds between a particular and its classes, both for processes and continuants) and **has_participant **(which holds between particular processes and particular continuants). I also adopt the convention of using boldface for relations involving particulars and italics for relations between types. I use lower case letters for variables for instances and italicized upper case for variables for classes, while I both italicize and capitalize terms for classes. In addition, I use the particular level relation **inheres_in**, which holds between individual instances of dependent and independent continuants [14]. It expresses a kind of one-sided ontological dependence, i.e. if p **inheres_in **x, then it is possible that × exists without p, but not that p exists without x. To avoid proliferation of relations I will stick to established relations when possible.

(1) $$\begin{array}{c} \text{M}\;is\text{\_}a\;\text{functional}\text{\_}\text{kind}:=\exists \text{F}(\text{F}\;is\text{\_}a\;\text{function}\;\wedge \;\text{M}\;has\text{\_}function\;\text{F}\wedge \\ \forall \text{x}\forall \text{f}\;\left(\text{f}\;\mathrm{instance}\text{\_}\mathrm{of}\;\text{F}\;\wedge \;\text{x}\;\mathrm{has}\text{\_}\mathrm{function}\;\text{f}\;\to \;\text{x}\;\mathrm{instance}\text{\_}of\;\text{M}\right) \end{array}$$

The specific function has to be assigned by way of definition to the specific kind in question. The classification of a mechanism as something which essentially has a specific function is necessary, but not sufficient. For a mechanism like photosynthesis to be what it is, the inner structure of the mechanism is also important, the material parts and their organization as well as the processes going on among them. So a mechanism has to have some substructure and functional organization. The explanatory value of a mechanism rests on this: The function of the whole is explained by the interplay of the functions of the parts. I will now try to capture these relations between a mechanism and its components as well as the relations between the components.

### Functional kinds and functional parts

It seems clear that the material components are parts of a mechanism, but the usual part-whole relation is too weak to capture the more specific relation of a mechanism to its components. More sophisticated subrelations taking into account granularity levels ([27] and the BioTop ontology [28,29]) do not help much either. A mechanism is clearly not a (homogeneous) collection of "grains". It is also doubtful whether the notion of a compound with components helps here, because of the "structure blindness" of the compound/component relation. Furthermore, the working parts can belong to different granularity levels: Pumped H^+ ^ions are (almost) elementary particles whereas the thylakoid membrane is on the level of cellular components. (This "level crossing" seems more the rule than the exception in cellular biology, cf. [10]). Mainly the functional role matters. So my suggestion is to introduce a relation "functional_part_of": a component must be a proper part of the whole and it must have a specified function. Similar suggestions have been made by Galton/Mizoguchi [15] and, Vieu/Aurnague [30], but the latter do not refer to processes explicitly and are more focused on properties of different parthood relations. The main point of difference here is that the functional parts for biological mechanisms are not automatically given as such by some previously available description, like a door handle as a functional part of a door [30]. Rather, the explanatory achievement of a mechanism is that the components, some biological entities like molecules, membranes etc. have certain functions that contribute to the function of the whole.

An approach that is somewhat close to my proposal is taken by Johansson et al. [31]. These authors claim that because most functions are relational and context-dependent with respect to a larger whole in which their bearers are imbedded, the problem of intrinsic functions can be avoided for the "constituent functions" of body parts and, one can add, of any subsystem of an organism. The functions of the parts are always relative to the whole. (For an organism as a whole the problem of intrinsic function re-appears, but that need not concern us here.) They also recognize multiple functions of one and the same body part.

(2) Xfunc_part_ofZ: = Xpart_ofZ∧∃f(finstance_ofFunction∧Xhas_functionf)

This seems still rather weak, because the ascription of functions is context-dependent as has been explained above. But the context is given by the mechanism we are interested in. It is *as part *of the mechanism m_WHOLE _that m has function x, not necessarily *per se*. (Note the analogy to the constituent functions in the functional anatomy approach.) For the relation of a functional part m to the mechanism as a whole m_WHOLE_, we want the following conditions to hold:

(1) m_WHOLE _is a mechanism, i.e. it is itself an instance of a functional kind with a specific function and realization process.

(2) m is a part of m_WHOLE_.

(3) The function of the part m contributes to the function of the whole mechanism m_WHOLE_.

That functions contribute to outcomes of larger structures like mechanisms would be definitional if we subscribe to the goal contribution account of functions mentioned above and in Johanssons approach it is definitional for constituent functions. It means that the (internal) realization process p_int _of the function of the part must contribute to the (external) process p_ext _enacted by m_WHOLE_. Galton and Mizoguchi [16] postulate a relation "p_int _contributes_to p_ext_" without specifying details. Johansson et al. [31] include a clause that explicitly refers to the larger anatomical structure in which the function-bearer performs its function. My suggestion is to use a parthood relation between the internal processes of the functional parts and the external process enacted by the whole. The BioTop ontology [28,29] contains the relation "has_processual_part" for expressing parthood between processes that seems useful here. As the same type of component may be a functional part in different types of mechanisms (H^+ ^ions or the cell membrane figure in many mechanisms), but mechanisms have their functional parts as necessary components, I will use the inverse relation **has_part**. Let us first define the contribution relation between functions f, f* indicating that the realization process of f is a part of the realization of f*:

(3) $$\begin{array}{c} \text{f}\;\mathrm{contributes}\text{\_}\mathrm{to}\;\text{f*:}\exists \text{r}\exists \text{p}\;\text{(f}\;\mathrm{has}\text{\_}\mathrm{realization}\;\text{r}\wedge \text{f*}\;\mathrm{has}\text{\_}\mathrm{realization}\;\text{p}\\ \wedge \text{p}\;\mathrm{has}\text{\_}\mathrm{processual}\text{\_}\mathrm{part}\;\text{r)} \end{array}$$

Note that this does not imply that every processual part of the encompassing process is a realization of a contributing function. There may well be "side effects" as the generation of heat, noise etc. will often be a concomitant result of the execution of the function without being identical to the realization as discussed above. But all realizations of the contributing functions must clearly be processual parts of the larger process enacted by the whole. It is to be hoped that this notion of contribution can be refined in future work. If an internal process p_int _is a processual part of the realization process p_ext _of m_WHOLE_, than the participant m of p_int _participates as well in p_ext _(contra Galton/Mizoguchi). It follows immediately from the definition of functions that m and m_WHOLE _participate in the realization processes of their respective functions, so I will not mention that explicitly in the following:

(4) $$\begin{array}{c} \text{x}\;\mathrm{has}\text{\_}\mathrm{func}\text{\_}\mathrm{part}\;\text{z: = x}\;\mathrm{has}\text{\_}\mathrm{part}\;\text{z}\wedge \\ \exists \text{f}\exists \text{f*}\exists \text{r}\exists \text{P}\;\text{(z}\;\mathrm{has}\text{\_}\mathrm{function}\;\text{f}\wedge \text{X}\;\mathrm{has}\text{\_}\mathrm{function}\;\text{f*}\wedge \text{f*}\;\mathrm{has}\text{\_}\mathrm{realization}\;\text{r}\\ \wedge \text{f}\;\mathrm{contributes}\text{\_}\mathrm{to}\;\text{f*}\wedge \text{r}\;\mathrm{has}\text{\_}\mathrm{participant}\;\text{z)} \end{array}$$

Let us now come back to the question, what a mechanism is.

It is (1) a complex continuant ("structured biological entity" from BioTop would be an appropriate class) that has (2) a specified biological function that is essential for it, and that (3) necessarily has a substructure of functional parts with functions that contribute to the function of the whole. A list of all the functional parts will give a further ("internal") specification of the mechanism in addition to the definition by its (external) function. Additional structural, probably topological and geometrical restrictions could be added for specific mechanisms, but will not concern us here.

Most of what has been said so far is not restricted to biological entities. One can distinguish "artefactual" and "biological mechanisms" by demanding that the latter are biological entities. These do not even have to be disjoint classes as there are probably biological artefacts, like genetically modified bacteria. Still, the question then arises whether whole organs or even whole organisms should be considered as mechanisms. In principle they could, but usually mechanisms are supposed to explain a particular function or phenomenon on a sub-organismic level. One could therefore include clauses to exclude whole organs from being mechanisms. It seems straightforward to add such domain-specific restrictions by defining e.g. "molecular mechanism" as something that has necessarily some biomolecule among its functional parts (not just among its parts simpliciter), or to demand that the mechanism is part of some organ.

### Relations between material components and processes

So far I focused on the relation of the mechanism as a whole to its components. The continuant components and processes of a mechanism stand in the relation of participation to each other: p **has_participant **x [26]. This simply follows from the conception of a function and can usually be generalized to the type level, as many types of processes essentially involve specific types of participants: Electron transport trivially has at least one electron as participant. (Complications arising from the fact that often a collective of molecules participates in a subprocess will be ignored for now. This matters if a certain concentration is necessary for a process to happen [14].) With the functional parts analysis this yields the statement that the functional parts of a mechanism are participants of the processes that are the realizations of these functions:

(5) $$\begin{array}{c} (\text{X}\;func\text{\_}part\text{\_}of\;\text{Z}\wedge \text{X}\;has\text{\_}function\;\text{F}\wedge \text{R}\;is\text{\_}a\;\text{Process)}\to \text{(F}\;has\text{\_}realization\;\text{R}\to \text{R}\\ has\text{\_}participant\;\text{X)} \end{array}$$

A converse statement is usually not true as the tokens of the participants do not always participate in processes of type P (again obvious for electrons), but could be formulated using a relation relative to time: × *sometimes_participates_in *P [26]. The more interesting connections, though, arise from the fact that several components are changed by participating in one internal process and by the connections of the internal processes themselves. Although the temporal order of the internal processes is important, a generalization is difficult, because there may also be cyclical elements or many processes running parallel. And in many cases we do not have knowledge of the temporal order. More possible relations are suggested by Grenon and Smith [32], such as relations that express more specific relations than "has_participant" between continuants and processes like: "x initiates p", "x terminates p". For sake of parsimony it seems reasonable to restrict oneself to as few as possible, but this has to be decided in the modeling of the specific mechanism of interest.

## Conclusions

It has been argued that an ontological analysis of biological mechanisms needs both continuants as their material parts and occurents that represent the changes of these material components. The connection of these continuants and occurents is given by the functions that inhere in the material components and have the specific processes as their realizations. The parts of the mechanism as well as the mechanism as a whole are functionally identified and therefore closely linked to the processes they enact. To make this explicit a conception of functional parts and a relation of contribution between functions have been introduced. This is a step towards a systematic causal-ontological analysis of complex biological systems. The contributions of the functions of parts to the whole could be systematically collected on the basis of ontologies that contain the functions of important components of biological mechanisms like the Gene Ontology. My goal was to bring together the rather general account of mechanisms proposed by philosophers of biology and more rigorous considerations from formal biomedical ontology. Admittedly, some details are still in need of refinement and the analysis could be made more precise in several ways: The context-dependence of functional parts would have to be made more explicit and the relationship between "absolute" types like natural kinds and the context-dependent functional kinds should be explored. One could also try to capture differences like the distinction between "active" and "passive" components of a mechanism and between changes of already existing components and the generation of new components. Also connections with topological and geometrical relations should be explored, such as the specification of the spatial region the mechanism as a whole or salient components of it occupy and the respective boundaries of a mechanism, both with respect to its environment and those among the submechanism of a complex mechanism.

## Competing interests

The author declares that he has no competing interests.
